# A Simple and Accurate LC–MS/MS Method for the Determination of Indobufen in Human Plasma and Its Clinical Application

**DOI:** 10.1155/jamc/2232924

**Published:** 2026-07-28

**Authors:** Hongyan Ji, Haijun Qu, Qie Guo, Donghua Liu, Changli Xu, Xue Yang, Fanbo Jin, Wen Xu

**Affiliations:** ^1^ Department of Pharmacy, The Affiliated Hospital of Qingdao University, Qingdao 266003, Shandong, China, qdu.edu.cn; ^2^ Department of Cardiovascular Surgery, The Affiliated Hospital of Qingdao University, Qingdao 266003, Shandong, China, qdu.edu.cn

**Keywords:** CRRT, indobufen, LC–MS/MS, renal insufficiency

## Abstract

As an antithrombotic agent, indobufen is widely used in China. However, the relationship between drug concentration and therapeutic efficacy in specific patient populations remains unclear. Therefore, it is crucial to establish a method for quantifying indobufen concentrations in human plasma to guide dosage recommendations. An electrospray ionization (ESI) power supply was utilized in conjunction with multireaction monitoring (MRM) in positive ion mode. Protein precipitation was employed to extract indobufen from the plasma sample. Separation was carried out using a ChromCORE‐C18 column with a gradient elution of the mobile phase, which was composed of 0.1% formic acid in water (Solvent A) and methanol (Solvent B) at a flow rate of 0.3 mL/min. The typical standard curve equation was *Y* = 0.763*X* + 0.0986 (*r* = 0.9993), demonstrating a good linear relationship within the range of 0.5–30 μg/mL. All methodological outcomes complied with regulatory guidelines. In patients with moderate renal insufficiency, it is advisable to reduce the dosage to 100 mg once daily. For patients with severe renal insufficiency, the dosage should be reduced to 1/3–1/4 of the standard dose. For patients undergoing continuous renal replacement therapy (CRRT), indobufen can be gradually eliminated, and most patients do not require dose adjustment.

## 1. Introduction

As a new generation of antithrombotic drugs, indobufen shows both antiplatelet and anticoagulant effects and plays an important role in the prevention and treatment of cardiovascular and cerebrovascular diseases [1]. Due to the reversible inhibition of platelet cyclooxygenase‐1 (COX‐1), which minimally affects gastrointestinal mucosal vasodilation, indobufen presents a lower risk of bleeding and fewer gastrointestinal reactions compared to aspirin. Numerous studies have demonstrated that indobufen exhibits comparable efficacy and superior safety to aspirin in the treatment of acute coronary syndrome [2–4]. Currently, indobufen is widely utilized in China as an alternative therapeutic option for patients who are unable to tolerate aspirin owing to conditions such as gastrointestinal ulcers or gout.

Indobufen is almost completely absorbed when administered orally, reaching peak plasma concentrations approximately 2 h postadministration. When administered orally at doses of 100 and 200 mg twice daily, steady‐state peak plasma concentrations can reach 16.7 and 29.2 mg/L, respectively. The drug is primarily excreted via the kidneys, with approximately 75% eliminated in urine as glucuronic acid conjugates, while a smaller portion is excreted in its unchanged form. The elimination half‐life of indobufen in healthy individuals is 6–8 h [5, 6]. In elderly patients, this half‐life extends to 11.6–14.4 h, and in patients with moderate‐to‐severe renal impairment, it further extends to 33.1 h [7, 8].

It is important to highlight that the mechanism of antiplatelet or anticoagulant action of indobufen is dose‐dependent. Indobufen exerts its antiplatelet effects through reversible inhibition of platelet COX‐1, leading to reduced production of thromboxane B2 and subsequent inhibition of platelet aggregation induced by adenosine diphosphate, platelet‐activating factor, and epinephrine. This inhibition demonstrates a certain dose–response relationship, with higher doses resulting in more potent suppression of platelet aggregation [9, 10]. Recent studies have further revealed that nuclear receptors, particularly NR4A1, play a crucial regulatory role in platelet activation and thrombus formation, providing new mechanistic insights into the pharmacological regulation of platelet function [11]. In terms of its anticoagulant mechanism, indobufen inhibits the catalytic activity of platelet factor 3 and decreases plasma levels of factors I, II, V, VIII, and X, thereby affecting both intrinsic and extrinsic coagulation pathways as well as the fibrinolytic system [12]. Consequently, this leads to prolonged activated partial thromboplastin time, prothrombin time (PT), and thrombin time. Previous studies have demonstrated that a single oral dose of 100 mg of indobufen does not significantly impact platelet factor 3 activity, whereas a 300 mg dose markedly inhibits the catalytic activity of platelet factor 3 [13].

Indobufen is typically administered at doses of 100 mg twice daily for its antiplatelet effects and 200 mg twice daily for its anticoagulant effects. Given the unique relationship between the mechanism of action and dose–response, as well as the research on the pharmacokinetics and pharmacodynamics in special populations such as the elderly, those with renal insufficiency, and those undergoing continuous renal replacement therapy (CRRT), the dosing adjustments remain ambiguous. Previous analytical methods for indobufen quantification, such as the gas–liquid chromatography (GLC) method reported by Fuccella et al. [5], involved complex and time‐consuming derivatization procedures. Although the derivatization step can be performed in batch mode, taking 60–90 min per batch, coupled with a chromatographic run time of 20–30 min per sample, this workflow remains practically constrained in clinical settings. In practice, samples are typically analyzed in small batches (e.g., several samples per run) to facilitate timely reporting. Under such realistic conditions, the required derivatization process becomes a critical bottleneck, markedly diminishing analytical throughput and efficiency. As a result, conventional GLC is poorly suited for routine clinical use. To address these drawbacks, this study was designed to develop and validate a rapid, sensitive, and robust LC–MS/MS method for the quantification of indobufen in human plasma.

## 2. Materials and Methods

### 2.1. Chemicals and Reagents

Indobufen and indomethacin (internal standard [IS]) were procured from the China Institute for Pharmaceutical and Biological Products Inspection (Beijing, China), with a certified purity of 99.9%. Formic acid and methanol were sourced from Anaqua Chemicals Supply (Shanghai, China), while drug‐free plasma for analysis and validation was supplied by the Affiliated Hospital of Qingdao University (Qingdao, China).

### 2.2. Instrumentation and LC–MS/MS Conditions

The LC system consisted of an Agilent 1290 liquid chromatograph (Agilent Technologies, California, USA) and an API 4000 triplet equipped with a quadrupole mass spectrometer (Applied Biosystems, California, USA) using an electrospray ionization (ESI) source. Chromatographic separation was performed on a ChromCORE‐C18 column (2.1 × 100 mm i.d., 3 μm; NanoChrom Technologies, Suzhou, China) at 40°C. The mobile phase consisted of 0.1% formic acid (Solvent A) and methanol (Solvent B), with a flow rate of 0.3 mL/min. The gradient program was set as follows: 0–0.5 min, 85% A; 0.5–1.5 min, 85%–20% A; 1.5–3 min, 20% A. The injection volume was 5 μL, and the autosampler was set at 40°C.

Mass spectrometer detection was conducted in the positive ionization mode with multiple reaction monitoring. The optimized parameters were as follows: ion spray voltage, 5500 V; source temperature, 550°C; curtain gas, 29 psi; nebulizer gas (ion source gas 1), 80 psi; heater gas (ion source gas 2), 80 psi; collision gas, 12 psi. Analyst Instrumental Control and Data Processing Software (Version 1.7.2, AB Sciex) and MultiQuant Software (Version 3.0.3, AB Sciex) were used for system operation and data analysis, respectively. The optimized parameters of the analytes and IS are shown in Table 1.

**TABLE 1:** Mass spectrometry settings for the quantification of indobufen and indomethacin.

Analytes	Indobufen	Indomethacin
PI	296.2	358.3
DI	250.2	139.1
DP (V)	99	75
CE (eV)	75	22

Abbreviations: CE, collision energy; DI, daughter ion; DP, declustering potential; PI, parent ion.

### 2.3. Stock Solutions and Working Solutions Preparation

Indobufen and indomethacin were weighed and dissolved in methanol, and the concentrations of the stock solutions were 1000 and 100 μg/mL, respectively. The stock solutions were serially diluted with a 50% methanol–water (v/v) solution. The concentrations of the calibration curve working solutions were set at 300, 200, 100, 50, 30, 10, and 5 μg/mL. The concentration of the IS working solution was maintained at 25 μg/mL. Subsequently, the calibration curve working solutions were diluted with blank plasma to achieve final concentrations of 30, 20, 10, 5, 3, 1, and 0.5 μg/mL.

Quality control (QC) working solutions of indobufen were prepared at concentrations of 250, 75, 15, and 5 μg/mL and further diluted with blank plasma to obtain QC solutions with final concentrations of 25, 7.5, 1.5, and 0.5 μg/mL, respectively.

### 2.4. Sample Preparation

Each plasma sample (100 μL) was spiked with 10 μL IS solution, and the mixture was vortexed for 30 s. Subsequently, 300 μL of methanol was added to precipitate proteins. The mixture was vortexed for 2 min and centrifuged at 12,000 rpm and 4°C for 10 min. The supernatant was diluted threefold with the initial mobile phase and then 50 μL was transferred to the insert of the autosampler vial and injected for LC–MS/MS analysis.

### 2.5. Method Validation

This method was validated according to the US Food and Drug Administration (FDA) guidelines for bioanalytical method validation [14], following the general validation strategy and acceptance criteria previously described by Zhao et al. for a multiplex LC–MS/MS method [15]. A recent study by Lou et al. demonstrated a similar LC–MS/MS approach for quantifying drugs in human plasma, providing a methodological framework for clinical bioanalytical applications [16].

#### 2.5.1. Selectivity

Plasma samples from six different volunteers were used as the matrix to mitigate the interference of potential confounding components on indobufen and IS. The response of the blank samples at the retention times of the analytes and IS should be less than 20% of the lower limit of quantification (LLOQ) for the analyte and less than 5% for the IS.

#### 2.5.2. Linearity and LLOQ

The calibration linearity range was designed based on the therapeutic window of indobufen. The calibration curve regression equation was generated by plotting the peak area ratio (Y) of indobufen to the IS against the concentration of indobufen (X) using the least squares method. Linearity was evaluated by analyzing the calibration curves at each concentration level in three independent batches. During the measurement process, the minimum concentration is set as LLOQ.

Compared to the interference component of the blank matrix, the intensity of LLOQ should be at least tenfold greater, ensuring a signal‐to‐noise ratio (S/N) of ≥ 10. The deviation of the LLOQ should be within ±20%, and the deviation of the samples on the calibration curve should be within ±15%.

#### 2.5.3. Precision and Accuracy

Five replicate samples of four QCs were analyzed to evaluate interday precision and accuracy across three independent batches, while intraday precision and accuracy were determined in a single analytical run. Precision is defined as the coefficient of variation (CV = standard deviation/mean × 100) that quantifies the degree of closeness of repeated measurements of an analyte; the CV should be within 15%, except for LLOQ (< 20%). Accuracy was determined as the percentage difference between the concentration of the QC level and its spiked value, which should be within 15%, except for LLOQ (< 20%).

#### 2.5.4. Dilution Integrity

The calibration curve range was established based on the therapeutic window of typical patients. However, in clinical practice, individual variability among patients can lead to exceptionally high values. To investigate abnormally high values, blank plasma was spiked with analyte at a concentration twofold above the upper limit of quantification (ULOQ). Serial twofold dilutions were subsequently performed on the spiked plasma for six iterations. The measured concentrations were then compared against the theoretical values. Finally, we compared the measured values against the theoretical values. The resulting accuracy and precision were then compared against expected outcomes, ensuring they remained within ±15%.

#### 2.5.5. Extraction Recovery and Matrix Effect

Human blank plasma from various sources was selected. After protein precipitation with methanol, the supernatant was collected and dried using nitrogen gas to obtain the blank matrix. The extraction recovery rate was assessed by comparing the analyte peak area of QCs spiked before extraction with the same QCs spiked after extraction. The matrix effect was determined by comparing the ratio of the peak area of the analyte in the presence of the matrix to that in the absence of the matrix for QCs. Both the assessment of the matrix effect and the extraction recovery rate were represented by the CV, with a requirement that the CV should be controlled within 15%.

#### 2.5.6. Analyte Stability

Six samples at each of three QC levels (25, 7.5, and 1.5 μg/mL) were analyzed to evaluate short‐term stability (stored at room temperature for 6 h), long‐term stability (stored at −80°C for 28 days), freeze–thaw stability (three cycles of freezing at −80°C followed by thawing at room temperature), and postpreparative stability (stored in the autosampler of the high‐performance liquid chromatography system for 12 h). The results need to comply with the requirement that the average analytical value is within ±15% of the spiked value.

### 2.6. Method Application

This trial is a prospective, open‐label clinical observation study conducted at the Affiliated Hospital of Qingdao University. Patients aged 18 years or older who required antiplatelet aggregation therapy were included, while those with severe coagulation dysfunction or severe anemia were excluded. The specific dosage was determined by the attending physician. Maximal platelet aggregation ratio of arachidonic acid‐stimulated platelets(MAR_AA_) was measured using a continuous dynamic platelet count method 7 days after drug administration (instrument: platelet function analyzer from Jiangsu Yinnuo Technology Co., Ltd., China). Based on reference [17] and the reference intervals provided by the laboratory, MAR_AA_ ≥ 50% indicates ineffective therapy, whereas MAR_AA_ < 50% indicates effective therapy. The hemofilter utilized was the BLS812G model (Bellco S.r.l.), which featured a polyether sulfone membrane with an effective membrane area of 1.2 square meters.

Blood samples were collected in 2 mL ethylenediaminetetraacetic acid potassium salt (EDTA‐K2), centrifuged at 12,000 rpm for 10 min at 4°C, and stored at −80°C until analysis. For LC–MS/MS, samples were thawed and processed as described in Section 2.4.

## 3. Results

### 3.1. Method Development and Optimization

To acquire sharper peaks, shorter running time, and quicker detection speed, the chromatographic conditions, various mobile phases, chromatography columns, and column temperatures were optimized.

**FIGURE 1:**
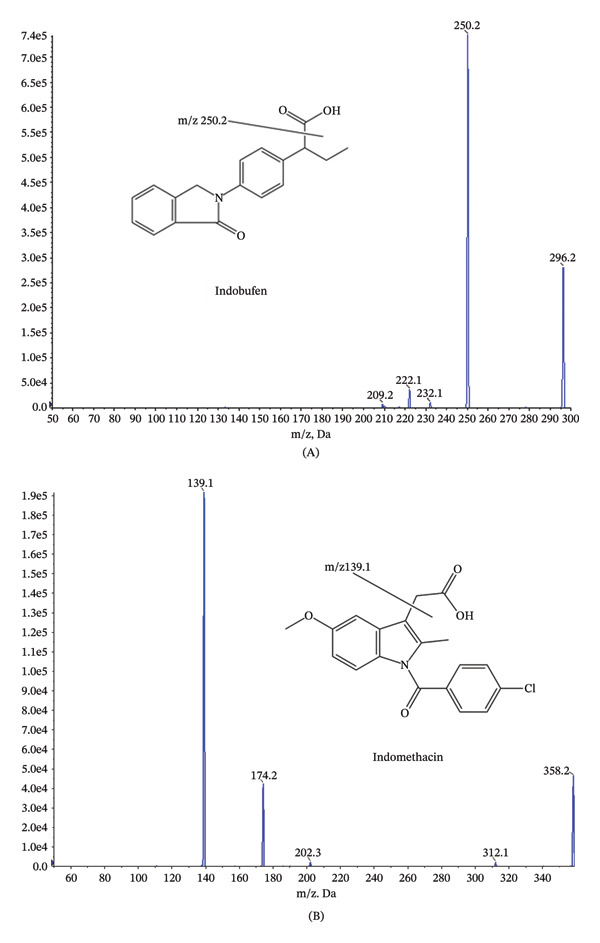
Chemical structures and product ion mass spectra of indobufen (A) and indomethacin ((B), internal standard).

To optimize the mass spectrometry (MS) conditions, the analyte was initially scanned using both ESI positive and negative ion modes, and the corresponding parameters are presented in Table 1. The chemical structures and product ion mass spectra of indobufen and indomethacin are shown in Figure 1. With regard to the stability of the method, the ChromCORE‐C18 column (2.1 × 100 mm, 3.0 μm), the Morphling column (2.1 × 100 mm, 3 μm), and the Agilent ZORBAX‐C18 column (2.1 × 150 mm, 5 μm) were compared, and discovered that all three columns exhibited good chromatographic resolution efficiency for the analyte.

Additionally, the composition of the mobile phase (0.1% formic acid and methanol) and the column temperature (30°C, 40°C, and 50°C) were optimized. When the mobile phase was fixed, the peaks of the analyte and IS overlapped, and the retention time was excessively long. Nevertheless, when the column temperature was set at 40°C and the mobile phase was changed to a gradient elution, the separation efficiency, retention time, and peak shape were all satisfactory. Given that the protein precipitation method has a straightforward extraction process that is convenient for daily operation and that methanol as a precipitant demonstrates better extraction efficiency than acetonitrile, this method was selected for the extraction of the analyte.

### 3.2. Method Validation

#### 3.2.1. Selectivity and Specificity

The representative chromatogram of indobufen and the IS are depicted in Figure 2. Analysis of blank plasma samples extracted from six distinct sources revealed that the endogenous interference intensity at the retention times of indobufen and IS was less than 20% of the LLOQ and 5% of the IS, respectively. Consequently, the method demonstrated adequate selectivity. Furthermore, the results indicated an absence of mutual signal interference between the analytes.

FIGURE 2Typical chromatogram of indobufen in the plasma. (A1) Blank plasma sample (indobufen); (B1) blank plasma sample (internal standard). (A2) LLOQ of indobufen; (B2) internal standard (LLOQ). (A3) indobufen plasma sample from patient; (B3): internal standard (patient plasma).

**Figure:**
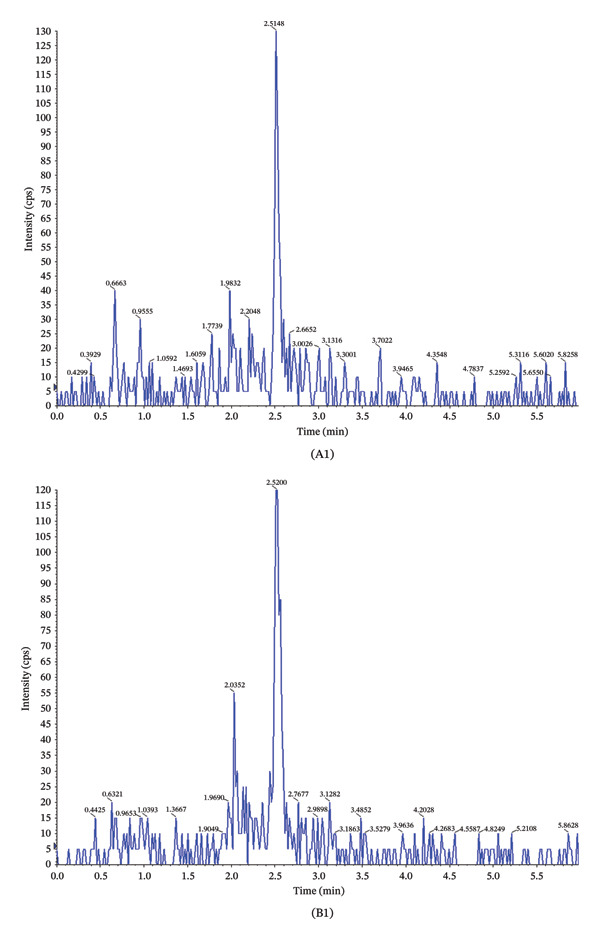

**Figure:**
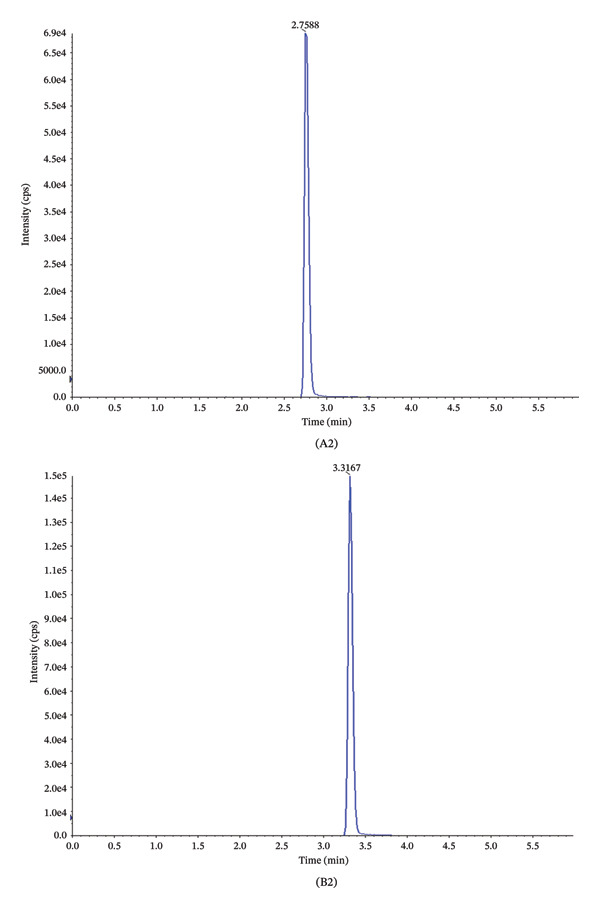

**Figure:**
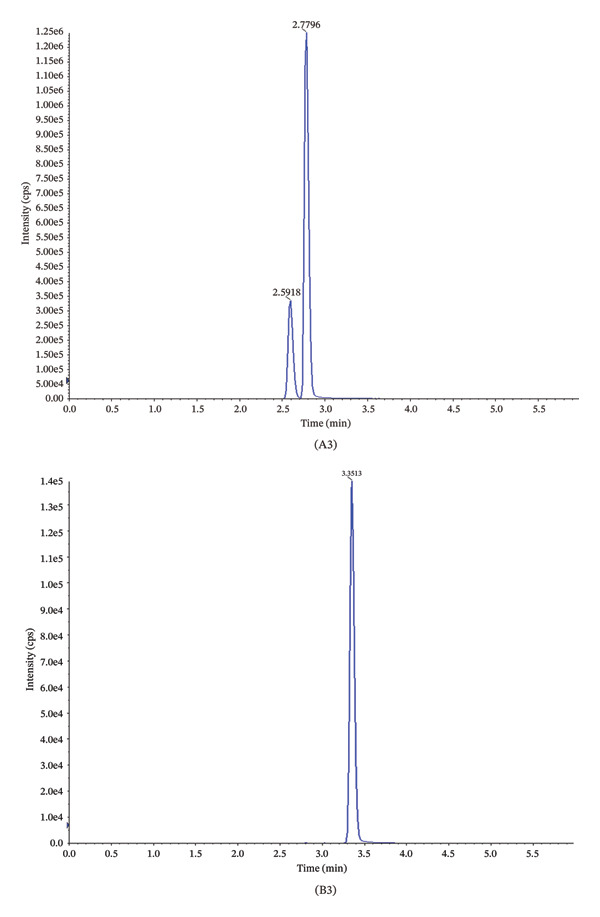

#### 3.2.2. Linearity and LLOQ

Within the concentration range of 0.5–30 μg/mL, the instrument response exhibits a linear relationship with the concentration of indobufen. The calibration curve was described by the equation *Y* = 0.763*X* + 0.0986 (*r* = 0.9993). The deviations of the measured concentrations from the spiked concentrations at each calibration level met the acceptance criteria of ±20% with CVs ≤ 8.12% (Table 2). These results indicated satisfactory linearity across a wide linear range.

**TABLE 2:** Accuracy and precision of methods for analyzing indobufen plasma drug concentration.

Spiked conc. (μg/mL)	Determined conc. (μg/mL) (mean ± SD)	Accuracy (%)	Precision (CV) (%)
Intraday (*n* = 5)			
0.5	0.50 ± 0.038	100.60	7.69
1.5	1.45 ± 0.033	96.53	2.31
7.5	7.43 ± 0.091	99.04	1.23
25	24.26 ± 0.95	97.04	3.90
Interday (*n* = 3)			
0.5	0.48 ± 0.039	96.31	8.12
1.5	1.46 ± 0.038	97.47	2.63
7.5	7.32 ± 0.21	97.65	2.88
25	23.36 ± 0.95	93.44	4.08

#### 3.2.3. Accuracy and Precision

The intraday and interday accuracy and precision of the indobufen determination methods are summarized in Table 2. For QCs, the intraday and interday accuracy ranged from 93.44% to 100.60%, while the intraday and interday precision ranged from 1.23% to 8.12%, with CVs ≤ 4.08%, all of which complied with the requirements of the guidelines.

#### 3.2.4. Extraction Recovery and Matrix Effect

The matrix effect and extraction recovery results for the indobufen assay are summarized in Table 3. The matrix effect was within the range of 98.35%–102.90%, and the extraction recovery was between 92.8% and 102.10%, with CVs ≤ 8.28%.

**TABLE 3:** Matrix effect and extraction recovery results of the indobufen concentration analysis method.

Spiked conc. (μg/mL)	Matrix effect (%)	CV (%)	Recovery (%)	CV (%)
1.5	102.90	3.95	102.10	3.51
7.5	98.35	4.05	92.80	8.28
25	98.36	2.01	99.50	3.73

#### 3.2.5. Dilution Integrity

The precision and accuracy of dilution integrity were maintained within ±15%. These results demonstrated that using blank plasma for diluting plasma samples was both reliable and compliant with clinical requirements when the measured concentration exceeded the ULOQ.

#### 3.2.6. Stability

The stability results of the indobufen method are presented in Table 4. The QCs all met the determined concentrations within ±15% of the spiked concentrations in all four environments, with CVs ≤ 7.61%.

**TABLE 4:** Results of a stability study of indobufen blood concentration analysis methods.

Spiked conc. (μg/mL)	Short‐term stability		Long‐term stability		Postpreparative stability		Freeze–thaw stability	
	Accuracy (%)	CV (%)	Accuracy (%)	CV (%)	Accuracy (%)	CV (%)	Accuracy (%)	CV (%)
1.5	112.33	6.59	102.56	2.28	103.26	2.52	95.58	2.97
7.5	106.80	3.97	99.95	1.01	101.22	1.23	98.06	1.40
25	91.90	5.84	89.47	5.84	90.69	6.01	87.99	7.61

### 3.3. Application to Patients

Case 1: A 62‐year‐old male patient weighing 70 kg presented with a creatinine level of 74 μmol/L and a creatinine clearance rate (CCr) of 90.2 mL/min. He was diagnosed with acute myocardial infarction and had a documented intolerance to aspirin. Indobufen was administered at a dose of 100 mg every 12 h. On the third day of treatment, the predose drug concentration was measured at 7.5 μg/mL, while the concentration 2.5 h postdose was 15.4 μg/mL. The measured MAR_AA_ value was 39%.

Case 2: A 68‐year‐old female weighing 64 kg was administered 100 mg of indobufen every 12 h following coronary artery bypass grafting (CABG). Her baseline renal function was characterized by a serum creatinine level of 128 μmol/L and CCr of 37.4 mL/min. On the fourth day of treatment, the predose plasma concentration was 27.7 μg/mL, while the concentration 2 h postdose increased to 36.0 μg/mL. The MAR_AA_ value was 31%. PT was mildly prolonged to 16.5 s, with an international normalized ratio (INR) of 1.32. No signs of bleeding were noted.

Case 3: A 93‐year‐old female weighing 55 kg with a creatinine level of 197 μmol/L and CCr of 13.6 mL/min, diagnosed with chronic kidney disease and unstable angina pectoris, was administered indobufen 100 mg every 12 h as antiplatelet therapy. On the fourth day, prior to drug administration, the concentration was 49.3 μg/mL, with a MAR_AA_ value of 19%. Both partial thromboplastin time and PT were slightly prolonged. No signs of bleeding were observed.

Case 4: A 61‐year‐old male patient weighing 75 kg with a history of gastric ulcer was administered indobufen 100 mg every 12 h for antiplatelet therapy, 6 h following CABG. On the second postoperative day, the patient developed acute renal insufficiency, anuria, creatinine 325 μmol/L, and received continuous treatment with CRRT at a therapeutic dose of 20–25 mL/kg/h. On the fourth day of CRRT treatment, preadministration indobufen concentration was measured at 18.9 μg/mL, which increased to 23.2 μg/mL after 2.5 h. The MAR_AA_ was 11.41%, and no signs of bleeding were observed.

Case 5: A 68‐year‐old male patient weighing 77 kg was administered indobufen 100 mg twice a day (at 7:30 a.m. and 5:00 p.m.) for antiplatelet therapy after CABG. On the second postoperative day, the patient exhibited anuria, elevated lactate levels, and a creatinine level of 223 μmol/L. CRRT was initiated at a therapeutic dose of 20–25 mL/kg/h. The drug concentration was measured at 6.8 μg/mL on the first day of CRRT treatment at 15:30, 10.01 μg/mL on the second day at 10:00, and 13.4 μg/mL on the same day at 20:50. On the fourth day, the concentration was 23.4 μg/mL at 10:00 and 18.3 μg/mL at 16:30. The MAR_AA_ was 22%, and no blood loss was observed during CRRT.

Case 6: A 60‐year‐old male patient weighing 62 kg with a history of gout was treated with indobufen 100 mg q12 h antiplatelet therapy after CABG. On the third day, the patient developed acute renal insufficiency with a creatinine level of 375 μmol/L, leading to an adjustment of the indobufen dosage to 100 mg once daily. On the fourth day, creatinine levels reached 446 μmol/L, concurrent with acute heart failure and anuria. As a result, indobufen administration was discontinued, and CRRT was initiated at a therapeutic dose of 20–25 mL/kg/h. Prior to CRRT, the concentration of indobufen was 45.6 μg/mL, with MAR_AA_ 41.60%. Following 20, 24, 28, and 32 h of continuous CRRT treatment, the indobufen concentrations were recorded as 28.7, 24.3, 21.4, and 18.9 μg/mL, respectively.

Independent sample reanalysis (ISR) was performed according to FDA guidelines. The results met the acceptance criteria, confirming the reproducibility of the method in real patient samples.

Pearson correlation analysis revealed no significant linear relationship between indobufen plasma concentration and MAR_AA_ when all six patients were included (*r* = 0.032, *p* = 0.95). Notably, Case 6 appeared to be an outlier. After excluding this case, a moderate negative correlation was observed in the remaining five patients (*r* = −0.407, *R* ^2^ = 0.166), suggesting a trend toward greater antiplatelet effects at higher plasma concentrations. However, this correlation did not reach statistical significance (*p* = 0.50). The discrepancy between the two analyses indicates substantial interindividual variability in the pharmacodynamic response to indobufen, which may be attributed to factors such as platelet count and function, mRNA expression of drug‐metabolizing enzymes, and genetic polymorphisms of the COX gene. Notably, recent studies have demonstrated that nuclear receptors such as NR4A1 play a critical regulatory role in platelet activation and thrombus formation, suggesting that variability in these signaling pathways may also contribute to the differential pharmacodynamic responses observed in our patients [11].

## 4. Discussion

In this study, a rapid, sensitive, and robust LC–MS/MS method was developed and validated for quantifying indobufen in human plasma. Unlike the conventional GLC approach described by Fuccella et al. [5], which requires a laborious derivatization step, our protocol employs a straightforward protein precipitation procedure, enabling efficient chromatographic separation with a shorter retention time and substantially reduced sample processing time, while maintaining the analytical accuracy required for clinical therapeutic drug monitoring.

All stability assessments met the FDA acceptance criteria (85%–115%). The accuracy for short‐term stability at the low QC level (1.5 μg/mL) was 112.33%, approaching the upper limit, whereas that for freeze–thaw stability at the high QC level (25 μg/mL) was 87.99%, nearing the lower boundary. These marginal deviations may be attributed to minor solvent evaporation, matrix effects at lower concentrations, degradation following repeated freeze–thaw cycles, or random interassay variability. Nevertheless, all values remained within the predefined acceptance range, and future methodological refinements—such as reducing freeze–thaw cycles or using certified reference materials—may further enhance assay robustness.

In patients with moderate to severe renal insufficiency, the plasma concentration of indobufen is markedly elevated, which can trigger anticoagulant effects. Accordingly, we recommend the following dose adjustments: (1) for moderate renal impairment, reduce to 100 mg once daily; (2) for severe renal impairment, decrease to one‐third or one‐quarter of the standard dose. Notably, in CRRT patients, indobufen undergoes partial clearance through the system. To maintain its antiplatelet effects, a dose of 100 mg every 12 h is recommended under appropriate monitoring.

Several limitations of this study should be acknowledged. First, indobufen is administered as a racemic mixture of the R‐ and S‐enantiomers, which exhibit distinct pharmacological activities and pharmacokinetic profiles. Our method quantified total indobufen without chiral separation, potentially obscuring enantiomer‐specific concentration–effect relationships. The lack of a significant correlation between total indobufen concentration and MAR_AA_ in our small patient cohort (*n* = 6) could be partially attributable to this unresolved stereochemistry, as the unmeasured ratio of active to inactive enantiomers may vary considerably among individuals. Future studies employing chiral LC–MS/MS methods are therefore warranted to delineate the pharmacokinetics and pharmacodynamics of each enantiomer. Second, the small sample size limits the statistical power and generalizability of our findings, and no formal population pharmacokinetic/pharmacodynamic modeling or multivariate analysis was performed. Third, although indobufen is highly protein‐bound, free drug concentrations and sieving coefficients during CRRT were not measured in this study. Consequently, the observed decline in total drug concentrations during CRRT should be interpreted as a preliminary clinical observation rather than a definitive pharmacokinetic conclusion.

## 5. Conclusion

A rapid and reliable LC–MS/MS method for quantifying indobufen in human plasma has been developed and validated, with a simple protein precipitation protocol that is well‐suited for routine therapeutic monitoring. Clinical application in patients with varying degrees of renal function suggests that indobufen dosage should be adjusted according to renal function status.

Collectively, our results should be interpreted as preliminary and hypothesis‐generating. Given the small sample size and the unresolved stereospecific pharmacology of the racemic mixture, larger prospective studies incorporating free drug monitoring and chiral analysis are needed to validate our findings.

## Author Contributions

Hongyan Ji and Haijun Qu: methodology, investigation, writing–original draft, and writing–review and editing. Qie Guo, Donghua Liu, Changli Xu, and Xue Yang: methodology, investigation, and writing–review and editing. Fanbo Jin: conceptualization, supervision, and resources. Wen Xu: funding acquisition, project administration, and writing–review and editing.

## Funding

This work was supported by the Shandong Provincial Traditional Chinese Medicine Science and Technology Development Program (No. 2019‐0394), the Therapeutic Drug Monitoring Research Fund of the Shandong Medical Association (No. YXH2020ZX042), the Shandong Provincial Natural Science Foundation (No. ZR2022MH069), and The Beijing Ronghe Medical Development Foundation (Project No. RHYX‐KT‐20251121‐0072).

## Ethics Statement

The study conformed to relevant laws and the Helsinki Declaration and was approved by the Ethics Committee of Qingdao University Affiliated Hospital (ethics approval number: QYFYEC2023‐29). Before enrollment, each patient was informed of the study details, and informed consent was obtained.

## Conflicts of Interest

The authors declare no conflicts of interest.

## Data Availability

The data that support the findings of this study are available from the corresponding author upon reasonable request.
